# The Expression of HMGB1 in Bone Marrow MSCs Is Upregulated by Hypoxia with Regulatory Effects on the Apoptosis and Adhesion

**DOI:** 10.1155/2016/4598927

**Published:** 2016-12-06

**Authors:** Mei-Yun Tan, Cai-Dong Zhang, Bo Xia, Jiang Guo, Zhong-Wei Fan, Tian-Hao Wu, Sen Wang, Shao-Feng Liu, Li Deng, Xing Guo, Yong-Can Huang

**Affiliations:** ^1^Department of Bone and Joint Surgery, The Affiliated Hospital of Southwest Medical University, Luzhou 646000, China; ^2^Laboratory of Stem Cell and Tissue Engineering, State Key Laboratory of Biotherapy, West China Hospital, Sichuan University, Chengdu 610041, China; ^3^Department of Burns and Plastic Surgery, The Affiliated Hospital of Southwest Medical University, Luzhou 646000, China; ^4^Shenzhen Engineering Laboratory of Orthopaedic Regenerative Technologies, Orthopaedic Research Center, Peking University Shenzhen Hospital, Shenzhen, China; ^5^Shenzhen Key Laboratory of Spine Surgery, Department of Spine Surgery, Peking University Shenzhen Hospital, Shenzhen, China; ^6^Department of Orthopaedics and Traumatology, Li Ka Shing Faculty of Medicine, The University of Hong Kong, Pok Fu Lam 999077, Hong Kong

## Abstract

*Background and Aims*. Hypoxia regulates the survival of mesenchymal stem cells (MSCs) but the mechanism is unclear. In hypoxia, the level of high mobility group box 1 (HMGB1) was increased in many cells which may be involved in the regulation of cell biology. The aim is to determine whether hypoxia affects the expression of HMGB1 in bone marrow MSCs (BM-MSCs) and to investigate the role of HMGB1 in the apoptosis and adhesion.* Methods*. BM-MSCs were exposed to hypoxia (1% O_2_) and normoxia (20% O_2_) and the expression of HMGB1 was measured by RT-PCR and western blotting. The apoptosis and adhesion of BM-MSCs were evaluated after interfered by different concentrations of HMGB1.* Results*. Expression of HMGB1 in BM-MSCs showed a significant upregulation in hypoxia when compared to those in normoxia. The adhesion of BM-MSCs was increased by HMGB1 in a concentration-dependent manner; the apoptosis effect of HMGB1 depended on its concentrations: HMGB1 at low concentration (50 ng/mL) promoted the apoptosis of BM-MSCs while HMGB1 at high concentration (≥100 ng/mL) reduced this apoptosis.* Conclusions*. Hypoxia enhanced the expression of HMGB1 in BM-MSCs with influences on apoptosis and adhesion and this could have a significant effect on the regenerative potential of MSC-based strategies.

## 1. Introduction

Mesenchymal stem cells (MSCs) are cells with the capacity of self-renewal and multiple differentiation abilities [1]. With the use of particular inducers, they can differentiate into osteocytes [2], chondrocytes [3], and other cells [4]. They are currently the most promising seed cells for tissue regeneration, having been successfully tested in treatment of many diseases such as degenerative diseases [5] and myocardial infarction [6] in humans. Nevertheless, the posttransplanted cells have to experience the hash microenvironment (hypoxia, low nutrition, inflammation, etc.) and most of them would die in the first 24 hours [7]. Quick loss of the implanted cells remains a big challenge for MSC-based therapies.

It was shown that hypoxic preconditioning could enhance the survival of MSCs after transplantation [8–10], but the underlying mechanism is still unknown. High mobility group box 1 (HMGB1), a nonhistone chromosomal binding protein, is released into extracellular space by damaged cells, necrotic cells, and activated inflammatory cells [11]. The biological function of these cells will be changed by HMGB1 with its subsequent binding to RAGE, toll-like receptors, and other membrane receptors [12, 13]. Studies have shown that hypoxia can induce the expression of HMGB1 in many kinds of cells such as chondrogenic ATDC5 cells, synovial fibroblast cells, monocyte/macrophage-lineage cell lines (HL-60 and U937) [14], and HTLA-230 cells [15]; interestingly, there was almost no expression in these cells under normoxic condition. Nevertheless, the effect of hypoxia on the expression of HMGB1 in BM-MSCs was still unclear. Thus, the first aim of this study was to investigate the expression level of HMGB1 in hypoxic-cultured BM-MSCs. Additionally, several researchers and our group have found that HMGB1 could significantly inhibit the proliferation and enhance the migration of BM-MSCs [16, 17]. But no finding regarding the regulation effects of HMGB1 in the apoptosis and adhesion of BM-MSCs was reported. Hence, the second aim of this work was to unravel the effect of HMGB1 on the apoptosis and the adhesive ability of BM-MSCs* in vitro*.

Therefore, in this study, BM-MSCs were isolated from rats and exposed to hypoxia; the expression of HMGB1 was then measured* in vitro *by RT-PCR and western blotting analysis. On the other hand, the effects of HMGB1 on the apoptosis and the adhesion of BM-MSCs were investigated by flow cytometry and Fibronectin-coating adhesives assessment, respectively.

## 2. Materials and Methods 

### 2.1. Isolation and Identification of BM-MSCs

Fifteen Sprague-Dawley rats weighing 150–200 g were supplied by the Experimental Animal Center of Southwest Medical University and the experimental procedures were approved by local Laboratory Animal Ethics Committee. BM-MSCs were isolated according to our previous description [18]. Briefly, rat bone marrow was collected by washing the femurs and tibias with Phosphate Buffered Saline (PBS) and seeded into flasks with Dulbecco's Modified Eagle's medium (DMEM) supplemented with 10% fetal bovine serum (FBS) and 1% antibiotic/antimycotic solution (Gibco). The bone marrow cells were cultured at 37°C in a humidified incubator with 5% CO_2_. To remove the nonadherent cells, the medium was replaced after 24 h and continued refreshing every 2-3 days. When the cells became 80%–90% confluent, they were subcultured at 1 : 2 dilution. In the flow cytometric analysis, the cells were identified with directly conjugated antibodies against anti-CD29, anti-CD31, anti-CD90, and anti-CD45 (BD Pharmingen, USA). BM-MSCs at passages 3–5 were used for all the experiments.

### 2.2. Experimental Hypoxic Condition

Hypoxic culture was performed when BM-MSCs of 3rd passages became 80%–90% confluence. The cells were incubated in hypoxia incubator chambers (Thermo, USA) at 37°C in the presence of 1% O_2_ and 5% CO_2_. Normoxic control was incubated in a humidified chamber containing 20% O_2_ and 5% CO_2_. To compare the differences of the expression of HMGB1 between hypoxic and normoxic cultures, cells cultured for 24 hours were harvested for RT-PCR and western blot analysis.

### 2.3. RT-PCR

Total RNA was extracted and one microgram of RNA was reverse-transcribed into cDNA using isoPLUS reagent (TAKARA, Japan). The expression of HMGB1 mRNA was analyzed by RT-PCR. In brief, the specific primer sequences of HMGB1 were designed and synthesized (Table 1) and the amplification conditions were listed as follows: denaturing at 95°C for 30 s followed by 40 cycles at 95°C for 15 s and 60°C for 30 s. Then the PCR reactions were separated by 1% agarose gel electrophoresis. All procedures were repeated three times in triplicate.

### 2.4. Western Blotting

Western blotting was performed to assess HMGB1 accumulation in BM-MSCs after being exposed to hypoxia. Whole-cell protein lysates were collected on ice. The protein (20 *μ*g) of each sample was separated on a 10% SDS-PAGE gel (Invitrogen, USA) and electrotransferred to nitrocellulose membranes (Amersham Pharmacia Biotech, USA). After the membranes were blocked in TBST containing 5% nonfat milk, the blots were incubated with HMGB1 primary antibody (Cell Signaling Technology, USA) (1 : 1000) overnight at 4°C. The immunoreactive bands were visualized using chemiluminescence regent kit (Sigma, USA) after the membranes incubated with HRP-conjugated secondary antibody. Signals from the bands were analyzed by an imaging densitometer (HP, USA). All procedures were repeated three times in triplicate.

### 2.5. Apoptosis Assessment

The method used for apoptosis assessment was similar to our previous description [19]. Briefly, 2 × 10^5^ cells were planted in 12-well plates and incubated for 24 h at 37°C incubator. Then, the initial solutions were replaced with serum-free DMEM for serum deprivation (SD) culture. HMGB1 with different concentrations (0, 10, 50, 100, and 200 ng/mL) were added at the same time after serum deprivation for cell apoptosis assays (*n* = 5, triplicate). After 24 hours of HMGB1 interfering, the cell apoptosis was assessed using the Annexin V-FITC apoptosis detection kit (BD Pharmingen, USA). Briefly, the cells were collected, washed, and resuspended in binding buffer; to stain the apoptotic cells, Annexin V-FITC and propidium iodide were added followed by 15 min reaction at room temperature. Then, the stained cells were subjected to flow cytometry using a FACScan flow cytometer (BD Biosciences, USA).

### 2.6. Cell Adhesion Assessment

The adhesion of BM-MSCs was evaluated according to previous report [8]. Briefly, Fibronectin (FN) (Prospec, Israel) with a concentration of 1 *μ*g/cm^2^ was used as a thin coating on 12-well plate surface overnight, and the supernatant solution was removed. BM-MSCs were seeded at 5 × 10^4^ cells/well in the 12-well plate, followed by 24 h cultivation with HMGB1 at different concentrations (0, 10, 50, 100, and 200 ng/mL) (*n* = 5). Cells seeded in the complete medium without FN were used as control. After 24 hours, the nonadherent cells were removed by washing with PBS for 30 seconds in the orbital shaker. Then the cells cohered to FN were counted in five random microscopic fields (100-fold magnification) in each well and the mean value of each well was measured to reflect the adhesion level. Experiments were repeated 3 times.

### 2.7. Statistical Analysis

Data were analyzed using SPSS 19.0 (SPSS 19.0 Inc., Chicago, IL, USA). One-way analysis of variance (ANOVA) followed by LSD-test was used to determine the significance of difference of all statistical data. Significance was accepted as *P* < 0.05.

## 3. Results

### 3.1. Characterization of BM-MSCs

**Figure 1:**
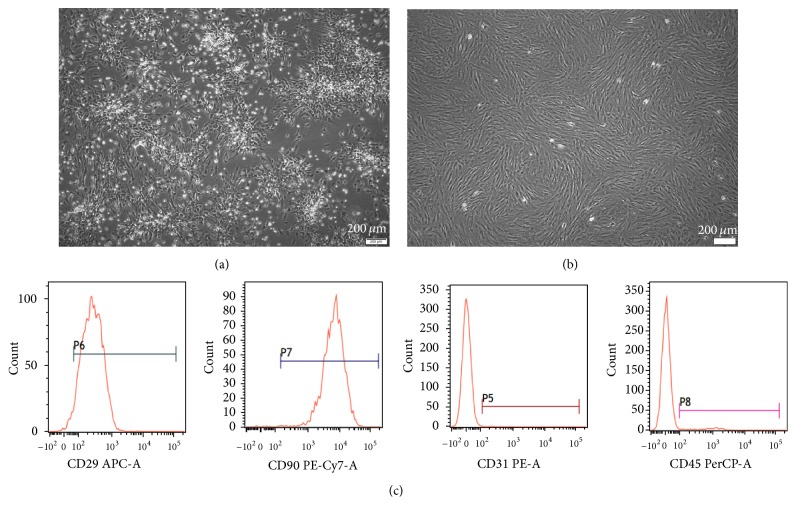
Characterization of BM-MSCs. (a) The P_0_ cells grew in colonies after being cultured for 5-6 days. (b) Representative images of P_3_ BM-MSCs (original magnification, ×40); the cells showed typically spindle-shape morphology. (c) Flow cytometric analysis of cell surface marker expression of the isolated cells. The cells were positive for MSCs specific markers CD29 and CD90 but negative for hematopoietic markers CD31 and CD45.

Cells were successfully isolated from the bone marrow. After 5-6 days of culture, the adherent cells grew in colonies (Figure 1(a)); cells at P_3_ (the third passage) showed a typical spindle-shaped appearance and arranged radially (Figure 1(b)). Flow cytometric analysis results indicated that the cells were positive for CD29 and CD90 (>95%) and negative for CD31 and CD45 (<5%) (Figure 1(c)).

### 3.2. Effect of Hypoxia on the Expression of HMGB1 in BM-MSCs

**Figure 2:**
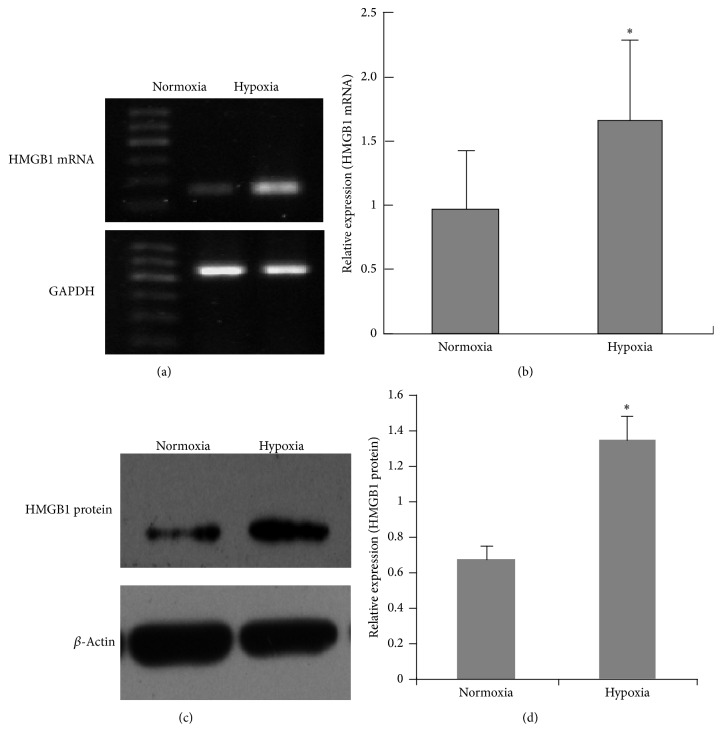
Effect of hypoxia on the expression of HMGB1 in BM-MSCs. Cells were exposed to hypoxic (1% O_2_) or normoxic conditions for 24 h. HMGB1 at mRNA and protein levels were detected by RT-PCR ((a)-(b)) and western blotting ((c)-(d)), respectively. GAPDH and *β*-actin were used as the control. The data was shown as mean ± SEM (*n* = 3). ^*∗*^ *P* < 0.05 versus normoxia.

RT-PCR data showed that 24-hour hypoxia exposure led to an obvious increase of the expression of HMGB1 mRNA in BM-MSCs when compared to cells in normoxia (*P* < 0.05) (Figures 2(a) and 2(b)). Consistent with the increase of mRNA levels, it was confirmed by western blotting experiment showing the higher levels of HMGB1 protein in BM-MSCs under hypoxia (*P* < 0.05) (Figures 2(c) and 2(d)). Hence, the expression level of HMGB1 in BM-MSCs was notably increased by hypoxia at gene and protein levels.

### 3.3. Effect of HMGB1 on the Apoptosis of BM-MSCs

**Figure 3:**
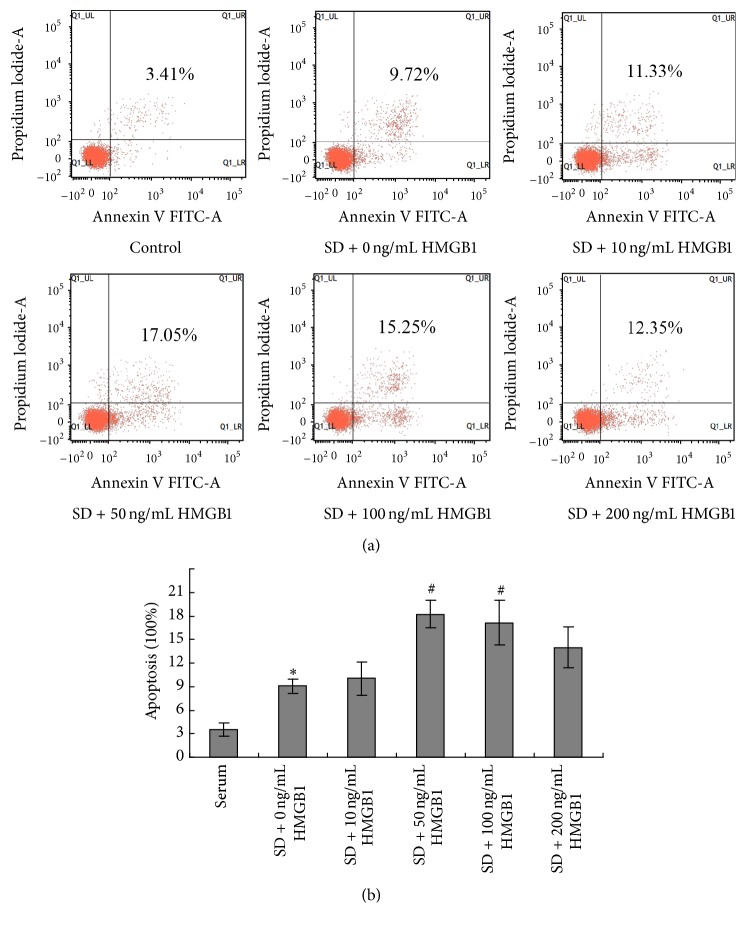
Effect of HMGB1 on the apoptosis of BM-MSCs. (a) Cells with different concentration of HMGB1 (0, 10, 50, 100, and 200 ng/mL) were exposed to serum deprivation (SD) for 24 hours. Apoptotic rate was calculated by FACS analysis of Annexin V/PI staining and the representative images of apoptotic cell populations were illustrated. (b) Data was shown as mean ± SEM (*n* = 5). ^*∗*^ *P* < 0.05 versus serum(s) group. ^#^ *P* < 0.05 versus the group of cells exposed to SD with 0 ng/mL HMGB1.

MSCs cultured in the absence or presence of different concentrations of HMGB1 (10, 50, 100, and 200 ng/mL) were exposed to serum deprivation (SD) for 24 hours. The apoptotic rate was measured by FACS analysis using Annexin V/PI staining. Increased apoptosis rate of BM-MSCs was observed after HMGB1 treatment (Figure 3(a)). Compared with the single SD culture group (0 ng/mL HMGB1, 9.03 ± 0.93), the apoptotic rate of BM-MSCs was much higher when the HMGB1 concentrations were 50 ng/mL (18.23 ± 1.74) and 100 ng/mL (17.13 ± 2.89) (*P* < 0.05). However, the apoptotic rate was not changed at the HMGB1 treatment group with concentration of 10 ng/mL (10.02 ± 2.08) and 200 ng/mL (13.97 ± 2.62), when compared with the single SD group (Figure 3(b)).

### 3.4. HMGB1 Increased the Adhesion of BM-MSCs

**Figure 4:**
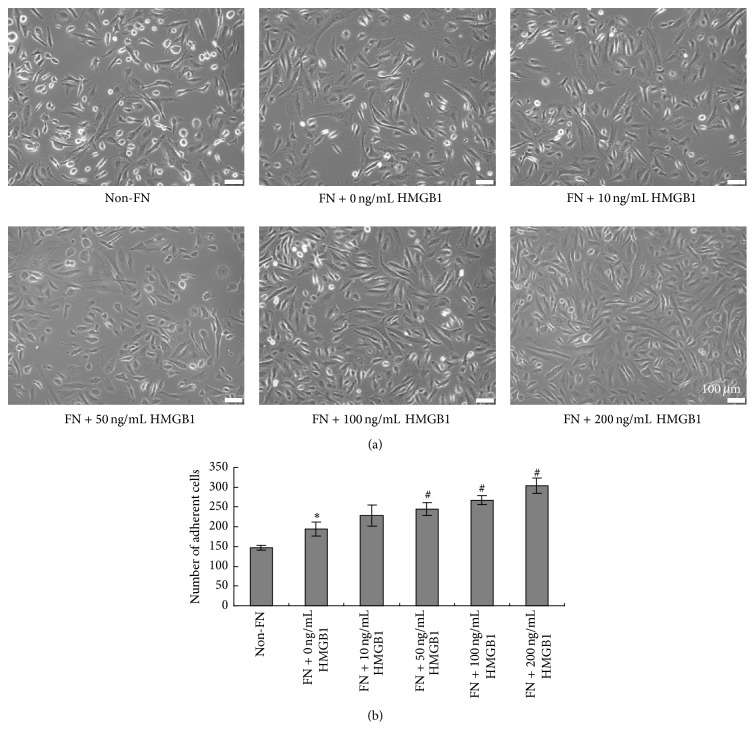
Effect of HMGB1 on the adhesion of BM-MSCs. (a) BM-MSCs were cultured with HMGB1 at the concentrations of 0, 10, 50, 100, and 200 ng/mL for 24 h; the representative images of BM-MSCs under phase-contrast microscopy (original magnification, ×100) were taken after the nonadherent cells were removed. (b) Quantification of adhesive cells. Data was shown as mean ± SEM (*n* = 5). ^*∗*^ *P* < 0.05 versus non-FN group. ^#^ *P* < 0.05 versus “FN + 0 ng/mL HMGB1” group.

To investigate the effects of HMGB1 on the adhesion of BM-MSCs, cells were seeded in 12-well plate which was coated with FN overnight, followed by 24 hours of 0–200 ng/mL HMGB1 treatments. With the increasing concentration of HMGB1, the number of adherent BM-MSCs was increased apparently (Figure 4(a)). Quantitative analysis showed that HMGB1 treatment groups (FN + 50 ng/mL: 245 ± 16.3; FN + 100 ng/mL: 267.6 ± 1.0; FN + 200 ng/mL: 304.0 ± 19.1) obviously increased the number of adhesive BM-MSCs when compared to single FN culture group (FN + 0 ng/mL, 194.4 ± 18.3) after 24 h treatment (*P* < 0.05) (Figure 4(b)).

## 4. Discussion 

This study provided evidence that hypoxia was able to induce significant upregulation of HMGB1 in BM-MSCs; then it further demonstrated that HMGB1 could promote the adhesion and regulate the apoptosis of BM-MSCs* in vitro*.

Oxygen concentration plays a crucial role in regulating the biology of BM-MSCs [20]. Previous studies show that hypoxia could influence the proliferation, migration, adhesion, differentiation, apoptosis, and angiogenesis ability of progenitor cells [8, 21, 22]. It is well known that HIF-1*α* is a key regulatory transcription factor during hypoxia [23], but researchers have never paid an equal attention to the effect of HMGB1 on MSCs under hypoxia. Recently, it was found that hypoxia or ischemia increased the levels of HMGB1 in many kinds of cells [14, 15]. Additionally, HMGB1 regulated the proliferation and migration of BM-MSCs [16, 17]. Considering these findings, we hypothesized that HMGB1 may also play an important role in regulating the behaviors of BM-MSCs in hypoxia. Hence, we compared the expression levels of HMGB1 in BM-MSCs under hypoxia and normoxia.

Many researches showed that HMGB1 conjugated with TLR or RAGE receptors of BM-MSCs which subsequently triggered the inflammatory reaction and vascular formation [12, 13, 24]. Nevertheless, little is known regarding its role in regulating biology of BM-MSCs. Cell adhesion is an important character for cell movement and tissue separation [25, 26]. It was reported that HMGB1 could improve the adhesion of endothelial cells [27, 28], bEnd3 cell line [29], and c-kit^+^ cells [30]. In this study, we further explored the effect of HMGB1 on the adhesion of BM-MSCs and concluded that HMGB1 was able to increase the adhesion of BM-MSCs in a concentration-dependent manner.

Furthermore, our study indicated that HMGB1 was able to accelerate the apoptosis of BM-MSCs, especially at the concentration of 50 ng/mL, but this increase tendency of apoptosis did not exist anymore when its concentration exceeded 100 ng/mL. It was pointed out that HMGB1 alone or together with hypoxia upregulated the expression of HIF-1*α* in BM-MSCs [16] and HIF-1*α* was helpful to protect BM-MSCs from serum deprivation induced apoptosis [31]. After HMGB1 (100 ng/mL) stimulation, the expression of HIF-1*α* mRNA and protein were notably increased in RA fibroblasts [32]. The higher concentration of HMGB1 may lead to higher expression level of HIF-1*α* against apoptosis in BM-MSCs, which may be a possible explanation for the phenomenon that 100 ng/mL did not induce higher apoptosis than the lower concentration of HMGB1 in our study. Hence, HMGB1 had the potential to accelerate the apoptosis of BM-MSCs, but it may also have a protective effect against cell apoptosis* via* the activation of HIF-1*α*.

Nevertheless, there are two major limitations that we should acknowledge in this work. First, the relationship between HIF-1*α* and HMGB1 in BM-MSCs was not well understood; future investigation using transgenic mouse model will be extremely meaningful to unravel this important issue. Second, further experiments are needed to understand the detailed mechanism concerning the regulation of HMGB1 in the adhesion and in the apoptosis of BM-MSCs. It can be presumed that there should be a HIF-1*α*-HMGB1 pathway to enhance the survival of postimplanted BM-MSCs. Definitely, the therapeutic potential of HMGB1 cannot be ignored, especially in the field of MSC-based therapies.

## Figures and Tables

**Table 1:** Specific primers used for real-time polymerase chain reaction.

Primer name	Primer sequences	Fragment length
HMGB1	Forward: 5′-GGCGGCTGTTTTGTTGACAT-3′	135 bp
	Reverse: 5′-ACCCAAAATGGGCAAAAGCA-3′	
GAPDH	Forward: 5′-ACCACAGTCCATGCCATCAC-3′	452 bp
	Reverse: 5′-TCCACCACCCTGTTGCTGTA-3′	
